# Iodido(1,10-phenanthroline-κ^2^ *N*,*N*′)(piperine-1-carbodithioato-κ^2^ *S*,*S*′)copper(II)

**DOI:** 10.1107/S1600536808028213

**Published:** 2008-09-06

**Authors:** Le-Qing Fan

**Affiliations:** a Institute of Materials Physical Chemistry, and Key Laboratory for Functional Materials of Fujian Higher Education, Huaqiao University, Quanzhou, Fujian 362021, People’s Republic of China

## Abstract

In the title compound, [Cu(C_6_H_10_NS_2_)I(C_12_H_8_N_2_)], the Cu^II^ ion is coordinated by one iodide ion, two N atoms of the phenanthroline ligand and two S atoms from the piperidyl­dithio­carbamate ligand in a distorted square-pyramidal environment.

## Related literature

For related literature, see: Englhardt *et al.* (1998 ▶); Fernández *et al.* (2000 ▶); Koh *et al.* (2003 ▶); Noro *et al.* (2000 ▶); Yaghi *et al.* (1998 ▶).
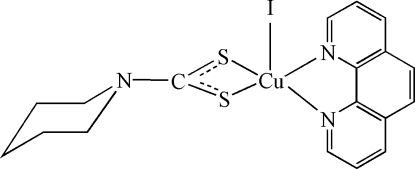


## Experimental

### 

#### Crystal data

[Cu(C_6_H_10_NS_2_)I(C_12_H_8_N_2_)]
*M* *_r_* = 530.91
Monoclinic, 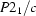

*a* = 6.408 (4) Å
*b* = 17.11 (1) Å
*c* = 18.400 (9) Å
β = 108.42 (2)°
*V* = 1914 (2) Å^3^
*Z* = 4
Mo *K*α radiation
μ = 2.98 mm^−1^
*T* = 293 (2) K
0.40 × 0.08 × 0.03 mm

#### Data collection

Rigaku Mercury CCD diffractometer
Absorption correction: multi-scan (*CrystalClear*; Rigaku,2000 ▶) *T*_min_ = 0.751, *T*_max_ = 0.915
14752 measured reflections
4236 independent reflections
3298 reflections with *I* > 2σ(*I*)
*R*_int_ = 0.069

#### Refinement

*R*[*F* ^2^ > 2σ(*F* ^2^)] = 0.063
*wR*(*F* ^2^) = 0.166
*S* = 1.09
4236 reflections
226 parameters
H-atom parameters constrained
Δρ_max_ = 1.41 e Å^−3^
Δρ_min_ = −1.52 e Å^−3^

### 

Data collection: *CrystalClear* (Rigaku, 2000 ▶); cell refinement: *CrystalClear*; data reduction: *CrystalClear*; program(s) used to solve structure: *SHELXS97* (Sheldrick, 2008 ▶); program(s) used to refine structure: *SHELXL97* (Sheldrick, 2008 ▶); molecular graphics: *SHELXTL* (Sheldrick, 2008 ▶); software used to prepare material for publication: *SHELXTL*.

## Supplementary Material

Crystal structure: contains datablocks global, I. DOI: 10.1107/S1600536808028213/im2082sup1.cif

Structure factors: contains datablocks I. DOI: 10.1107/S1600536808028213/im2082Isup2.hkl

Additional supplementary materials:  crystallographic information; 3D view; checkCIF report

## Figures and Tables

**Fig. 1.**
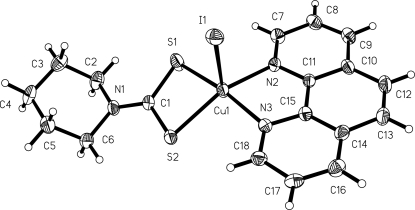
The molecular structure of (I) with 30% probability displacement ellipsoids (arbitrary spheres for H atoms).

## supplementary crystallographic information

### Comment

Research concerning transition metal complexes has been rapidly expanding because of their fascinating structural diversity, as well as their potential applications as functional materials and enzymes (Noro *et al.*, 2000; Yaghi *et al.*, 1998). Dialkyldithiocarbamate anions, which are typical sulfur ligands, acting as monodentate, bidentate or bridging ligands, are often chosen for the preparation of a considerable structural variety of complexes (Englhardt *et al.*, 1998; Fernández *et al.*, 2000; Koh *et al.*, 2003). I report here the crystal structure of the title copper(II) complex, (I), contanining a piperidyldithiocarbamate ligand.

The crystal structure of (I) is built of discrete molecules of the Cu^II^ complex (Fig. 1). The Cu^II^ ion is five-coordinated in a distorted square-pyramidal environment by one I atom in the apical position, two N atoms from the phenanthroline ligand and two S atoms from the piperidyldithiocarbamate ligand in the basal plane (Table 1).

### Experimental

A mixture of Cu(Ac)_2_.H_2_O (0.08 g, 0.4 mmol), NaS_2_CNC_5_H_10_.2H_2_O (0.09 g, 0.4 mmol), 1,10-phenanthroline (0.06 g 0.4 mmol) and NaI.2H_2_O (0.07 g, 0.4 mmol) was stirred in DMF (15 ml). 2-PrOH was diffused into the resulting solution, yielding single crystals of (I).

### Refinement

H atoms were positioned geometrically and refined as riding atoms, with C—H = 0.93 (aromatic) or 0.97 Å (piperidyl), *U*_iso_(H) = 1.2*U*_eq_(C).

### Crystal data

**Table:**

[Cu(C_6_H_10_NS_2_)I(C_12_H_8_N_2_)]	*F*(000) = 1044
*M**_r_* = 530.91	*D*_x_ = 1.843 Mg m^−^^3^
Monoclinic, *P*2_1_/*c*	Mo *K*α radiation, λ = 0.71073 Å
Hall symbol: -P 2ybc	Cell parameters from 4118 reflections
*a* = 6.408 (4) Å	θ = 3.3–27.5°
*b* = 17.11 (1) Å	µ = 2.98 mm^−^^1^
*c* = 18.400 (9) Å	*T* = 293 K
β = 108.42 (2)°	Prism, black
*V* = 1914 (2) Å^3^	0.40 × 0.08 × 0.03 mm
*Z* = 4	

### Data collection

**Table:**

Rigaku Mercury CCD diffractometer	4236 independent reflections
Radiation source: Sealed Tube	3298 reflections with *I* > 2σ(*I*)
Graphite Monochromator	*R*_int_ = 0.069
ω scans	θ_max_ = 27.5°, θ_min_ = 2.3°
Absorption correction: multi-scan (CrystalClear; Rigaku,2000)	*h* = −8→8
*T*_min_ = 0.751, *T*_max_ = 0.915	*k* = −22→22
14752 measured reflections	*l* = −23→23

### Refinement

**Table:**

Refinement on *F*^2^	Primary atom site location: structure-invariant direct methods
Least-squares matrix: full	Secondary atom site location: difference Fourier map
*R*[*F*^2^ > 2σ(*F*^2^)] = 0.063	Hydrogen site location: inferred from neighbouring sites
*wR*(*F*^2^) = 0.166	H-atom parameters constrained
*S* = 1.09	*w* = 1/[σ^2^(*F*_o_^2^) + (0.0831*P*)^2^ + 0.6436*P*] where *P* = (*F*_o_^2^ + 2*F*_c_^2^)/3
4236 reflections	(Δ/σ)_max_ < 0.001
226 parameters	Δρ_max_ = 1.41 e Å^−^^3^
0 restraints	Δρ_min_ = −1.52 e Å^−^^3^

### Special details

**Table:**

Geometry. All e.s.d.'s (except the e.s.d. in the dihedral angle between two l.s. planes) are estimated using the full covariance matrix. The cell e.s.d.'s are taken into account individually in the estimation of e.s.d.'s in distances, angles and torsion angles; correlations between e.s.d.'s in cell parameters are only used when they are defined by crystal symmetry. An approximate (isotropic) treatment of cell e.s.d.'s is used for estimating e.s.d.'s involving l.s. planes.
Refinement. Refinement of *F*^2^ against ALL reflections. The weighted *R*-factor *wR* and goodness of fit *S* are based on *F*^2^, conventional *R*-factors *R* are based on *F*, with *F* set to zero for negative *F*^2^. The threshold expression of *F*^2^ > σ(*F*^2^) is used only for calculating *R*-factors(gt) *etc*. and is not relevant to the choice of reflections for refinement. *R*-factors based on *F*^2^ are statistically about twice as large as those based on *F*, and *R*- factors based on ALL data will be even larger.

### Fractional atomic coordinates and isotropic or equivalent isotropic displacement parameters (Å^2^)

**Table:**

	*x*	*y*	*z*	*U*_iso_*/*U*_eq_	
Cu1	0.45983 (13)	0.35695 (5)	0.41693 (4)	0.0396 (2)	
I1	0.22830 (8)	0.21581 (3)	0.35611 (3)	0.04846 (19)	
S1	0.2502 (3)	0.45295 (13)	0.33902 (11)	0.0603 (6)	
S2	0.6695 (3)	0.39466 (10)	0.34223 (9)	0.0399 (4)	
N1	0.4499 (9)	0.5001 (3)	0.2390 (3)	0.0405 (12)	
N2	0.3528 (9)	0.3678 (3)	0.5106 (3)	0.0351 (11)	
N3	0.6887 (9)	0.2896 (3)	0.4912 (3)	0.0361 (12)	
C1	0.4559 (11)	0.4560 (3)	0.2983 (3)	0.0362 (14)	
C2	0.2686 (13)	0.5527 (4)	0.2018 (4)	0.0539 (19)	
H2A	0.3243	0.6054	0.2016	0.065*	
H2B	0.1644	0.5533	0.2303	0.065*	
C3	0.1534 (13)	0.5263 (4)	0.1201 (4)	0.0532 (18)	
H3A	0.0410	0.5641	0.0947	0.064*	
H3B	0.0819	0.4765	0.1207	0.064*	
C4	0.3171 (14)	0.5182 (5)	0.0754 (4)	0.062 (2)	
H4A	0.2430	0.4966	0.0251	0.074*	
H4B	0.3735	0.5694	0.0685	0.074*	
C5	0.5064 (13)	0.4654 (5)	0.1179 (4)	0.059 (2)	
H5A	0.6141	0.4637	0.0909	0.071*	
H5B	0.4522	0.4127	0.1197	0.071*	
C6	0.6142 (12)	0.4953 (4)	0.1989 (4)	0.0492 (17)	
H6A	0.7317	0.4602	0.2262	0.059*	
H6B	0.6774	0.5465	0.1973	0.059*	
C7	0.1837 (11)	0.4078 (3)	0.5183 (4)	0.0411 (15)	
H7A	0.1003	0.4386	0.4778	0.049*	
C8	0.1278 (12)	0.4048 (4)	0.5863 (4)	0.0472 (17)	
H8A	0.0098	0.4340	0.5905	0.057*	
C9	0.2441 (12)	0.3598 (4)	0.6453 (4)	0.0436 (16)	
H9A	0.2049	0.3568	0.6898	0.052*	
C10	0.4269 (12)	0.3172 (4)	0.6389 (4)	0.0399 (15)	
C11	0.4745 (10)	0.3239 (3)	0.5700 (3)	0.0328 (13)	
C12	0.5573 (13)	0.2666 (4)	0.6979 (4)	0.0483 (17)	
H12A	0.5235	0.2607	0.7432	0.058*	
C13	0.7278 (13)	0.2278 (4)	0.6880 (4)	0.0478 (17)	
H13A	0.8131	0.1963	0.7275	0.057*	
C14	0.7832 (11)	0.2334 (4)	0.6183 (4)	0.0416 (15)	
C15	0.6546 (11)	0.2818 (3)	0.5602 (4)	0.0343 (13)	
C16	0.9566 (13)	0.1938 (4)	0.6047 (4)	0.0488 (17)	
H16A	1.0493	0.1625	0.6426	0.059*	
C17	0.9907 (12)	0.2009 (4)	0.5348 (4)	0.0463 (16)	
H17A	1.1035	0.1734	0.5246	0.056*	
C18	0.8542 (11)	0.2500 (4)	0.4793 (4)	0.0408 (14)	
H18A	0.8795	0.2552	0.4325	0.049*	

### Atomic displacement parameters (Å^2^)

**Table:**

	*U*^11^	*U*^22^	*U*^33^	*U*^12^	*U*^13^	*U*^23^
Cu1	0.0419 (5)	0.0493 (5)	0.0317 (4)	0.0084 (3)	0.0172 (4)	0.0075 (3)
I1	0.0453 (3)	0.0554 (3)	0.0486 (3)	−0.0062 (2)	0.0204 (2)	−0.01413 (19)
S1	0.0582 (13)	0.0803 (13)	0.0535 (11)	0.0306 (10)	0.0335 (10)	0.0285 (10)
S2	0.0402 (9)	0.0471 (9)	0.0349 (8)	0.0043 (7)	0.0155 (7)	0.0071 (6)
N1	0.046 (3)	0.046 (3)	0.032 (3)	0.005 (2)	0.015 (3)	0.011 (2)
N2	0.038 (3)	0.038 (3)	0.028 (3)	0.000 (2)	0.010 (2)	−0.001 (2)
N3	0.035 (3)	0.043 (3)	0.032 (3)	0.001 (2)	0.012 (2)	0.000 (2)
C1	0.042 (4)	0.039 (3)	0.027 (3)	0.001 (3)	0.010 (3)	0.001 (2)
C2	0.068 (5)	0.045 (4)	0.042 (4)	0.014 (4)	0.010 (4)	0.009 (3)
C3	0.055 (5)	0.041 (4)	0.052 (4)	0.003 (3)	−0.001 (4)	0.004 (3)
C4	0.071 (6)	0.076 (5)	0.030 (4)	0.002 (4)	0.002 (4)	−0.001 (3)
C5	0.058 (5)	0.079 (5)	0.045 (4)	0.009 (4)	0.022 (4)	0.005 (4)
C6	0.045 (4)	0.060 (4)	0.041 (4)	0.001 (3)	0.011 (3)	0.018 (3)
C7	0.046 (4)	0.038 (3)	0.044 (4)	0.010 (3)	0.021 (3)	0.001 (3)
C8	0.051 (4)	0.050 (4)	0.051 (4)	0.001 (3)	0.029 (4)	−0.007 (3)
C9	0.058 (5)	0.042 (3)	0.037 (4)	−0.010 (3)	0.023 (4)	−0.011 (3)
C10	0.046 (4)	0.044 (3)	0.034 (3)	−0.009 (3)	0.019 (3)	−0.003 (3)
C11	0.036 (4)	0.032 (3)	0.029 (3)	−0.009 (2)	0.008 (3)	0.000 (2)
C12	0.059 (5)	0.056 (4)	0.031 (3)	−0.009 (3)	0.016 (3)	0.001 (3)
C13	0.055 (5)	0.050 (4)	0.034 (4)	−0.008 (3)	0.006 (3)	0.008 (3)
C14	0.039 (4)	0.041 (3)	0.040 (4)	−0.005 (3)	0.006 (3)	0.005 (3)
C15	0.037 (4)	0.033 (3)	0.033 (3)	−0.001 (2)	0.012 (3)	−0.001 (2)
C16	0.049 (5)	0.040 (3)	0.052 (4)	0.003 (3)	0.010 (4)	0.006 (3)
C17	0.034 (4)	0.046 (4)	0.058 (5)	0.006 (3)	0.012 (3)	−0.003 (3)
C18	0.035 (4)	0.046 (3)	0.043 (4)	0.002 (3)	0.014 (3)	−0.002 (3)

### Geometric parameters (Å, °)

**Table:**

Cu1—N3	2.020 (5)	C5—H5A	0.9700
Cu1—N2	2.055 (5)	C5—H5B	0.9700
Cu1—S2	2.2972 (19)	C6—H6A	0.9700
Cu1—S1	2.311 (2)	C6—H6B	0.9700
Cu1—I1	2.8694 (16)	C7—C8	1.408 (8)
S1—C1	1.711 (6)	C7—H7A	0.9300
S2—C1	1.711 (7)	C8—C9	1.348 (10)
N1—C1	1.317 (7)	C8—H8A	0.9300
N1—C2	1.457 (9)	C9—C10	1.416 (10)
N1—C6	1.466 (8)	C9—H9A	0.9300
N2—C7	1.326 (8)	C10—C11	1.398 (8)
N2—C11	1.353 (8)	C10—C12	1.433 (10)
N3—C18	1.334 (8)	C11—C15	1.419 (9)
N3—C15	1.361 (7)	C12—C13	1.339 (11)
C2—C3	1.520 (10)	C12—H12A	0.9300
C2—H2A	0.9700	C13—C14	1.438 (10)
C2—H2B	0.9700	C13—H13A	0.9300
C3—C4	1.530 (11)	C14—C16	1.391 (10)
C3—H3A	0.9700	C14—C15	1.397 (9)
C3—H3B	0.9700	C16—C17	1.376 (10)
C4—C5	1.517 (11)	C16—H16A	0.9300
C4—H4A	0.9700	C17—C18	1.395 (10)
C4—H4B	0.9700	C17—H17A	0.9300
C5—C6	1.520 (10)	C18—H18A	0.9300
			
N3—Cu1—N2	81.2 (2)	C4—C5—H5B	109.6
N3—Cu1—S2	97.36 (16)	C6—C5—H5B	109.6
N2—Cu1—S2	152.84 (15)	H5A—C5—H5B	108.1
N3—Cu1—S1	168.73 (16)	N1—C6—C5	109.6 (6)
N2—Cu1—S1	99.97 (15)	N1—C6—H6A	109.7
S2—Cu1—S1	76.38 (7)	C5—C6—H6A	109.7
N3—Cu1—I1	87.66 (15)	N1—C6—H6B	109.7
N2—Cu1—I1	97.75 (14)	C5—C6—H6B	109.7
S2—Cu1—I1	109.33 (6)	H6A—C6—H6B	108.2
S1—Cu1—I1	103.21 (8)	N2—C7—C8	121.5 (6)
C1—S1—Cu1	85.2 (2)	N2—C7—H7A	119.3
C1—S2—Cu1	85.6 (2)	C8—C7—H7A	119.3
C1—N1—C2	123.5 (6)	C9—C8—C7	120.4 (6)
C1—N1—C6	123.1 (5)	C9—C8—H8A	119.8
C2—N1—C6	113.1 (5)	C7—C8—H8A	119.8
C7—N2—C11	118.8 (5)	C8—C9—C10	119.2 (6)
C7—N2—Cu1	129.5 (4)	C8—C9—H9A	120.4
C11—N2—Cu1	111.6 (4)	C10—C9—H9A	120.4
C18—N3—C15	118.3 (6)	C11—C10—C9	117.2 (6)
C18—N3—Cu1	128.6 (4)	C11—C10—C12	119.6 (6)
C15—N3—Cu1	113.0 (4)	C9—C10—C12	123.2 (6)
N1—C1—S1	123.8 (5)	N2—C11—C10	122.9 (6)
N1—C1—S2	123.5 (5)	N2—C11—C15	117.6 (5)
S1—C1—S2	112.7 (3)	C10—C11—C15	119.6 (6)
N1—C2—C3	110.3 (6)	C13—C12—C10	120.1 (6)
N1—C2—H2A	109.6	C13—C12—H12A	120.0
C3—C2—H2A	109.6	C10—C12—H12A	120.0
N1—C2—H2B	109.6	C12—C13—C14	122.1 (7)
C3—C2—H2B	109.6	C12—C13—H13A	119.0
H2A—C2—H2B	108.1	C14—C13—H13A	119.0
C2—C3—C4	111.0 (7)	C16—C14—C15	117.5 (6)
C2—C3—H3A	109.4	C16—C14—C13	124.4 (7)
C4—C3—H3A	109.4	C15—C14—C13	118.0 (7)
C2—C3—H3B	109.4	N3—C15—C14	122.8 (6)
C4—C3—H3B	109.4	N3—C15—C11	116.5 (5)
H3A—C3—H3B	108.0	C14—C15—C11	120.6 (6)
C5—C4—C3	110.5 (6)	C17—C16—C14	119.8 (7)
C5—C4—H4A	109.6	C17—C16—H16A	120.1
C3—C4—H4A	109.6	C14—C16—H16A	120.1
C5—C4—H4B	109.6	C16—C17—C18	119.3 (6)
C3—C4—H4B	109.6	C16—C17—H17A	120.4
H4A—C4—H4B	108.1	C18—C17—H17A	120.4
C4—C5—C6	110.3 (6)	N3—C18—C17	122.2 (6)
C4—C5—H5A	109.6	N3—C18—H18A	118.9
C6—C5—H5A	109.6	C17—C18—H18A	118.9
